# MRI and Clinical Biomarkers Overlap between Glaucoma and Alzheimer’s Disease

**DOI:** 10.3390/ijms241914932

**Published:** 2023-10-05

**Authors:** Alessio Martucci, Francesca Di Giuliano, Silvia Minosse, Giulio Pocobelli, Carlo Nucci, Francesco Garaci

**Affiliations:** 1 Ophthalmology Unit, Department of Experimental Medicine, University of Rome “Tor Vergata”, 00133 Rome, Italy; alessio.martucci@live.it (A.M.); giulio.pocobelli@gmail.com (G.P.); 2 Neuroradiology Unit, Department of Biomedicine and Prevention, University of Rome “Tor Vergata”, 00133 Rome, Italy; francesca.di.giuliano@uniroma2.it; 3 Diagnostic Imaging Unit, Department of Biomedicine and Prevention, University of Rome “Tor Vergata”, 00133 Rome, Italy; silvia.minosse@ptvonline.it (S.M.); francesco.garaci@uniroma2.it (F.G.); 4 San Raffaele Cassino, 03043 Frosinone, Italy

**Keywords:** glaucoma, Alzheimer’s disease, magnetic resonance imaging, neuroimaging, clinical biomarkers, functional magnetic resonance, diffusion magnetic resonance

## Abstract

Glaucoma is the leading cause of blindness worldwide. It is classically associated with structural and functional changes in the optic nerve head and retinal nerve fiber layer, but the damage is not limited to the eye. The involvement of the central visual pathways and disruption of brain network organization have been reported using advanced neuroimaging techniques. The brain structural changes at the level of the areas implied in processing visual information could justify the discrepancy between signs and symptoms and underlie the analogy of this disease with neurodegenerative dementias, such as Alzheimer’s disease, and with the complex group of pathologies commonly referred to as “disconnection syndromes.” This review aims to summarize the current state of the art on the use of advanced neuroimaging techniques in glaucoma and Alzheimer’s disease, highlighting the emerging biomarkers shared by both diseases.

## 1. Introduction

Glaucoma is a multifactorial degenerative optic neuropathy characterized by the loss of retinal ganglion cells and consequent irreversible damage to the visual field. After cataracts, it is the second leading cause of blindness worldwide [1,2,3]. The complex nature of this disease makes its diagnosis sometimes difficult, which can lead to delays in treatment [1,4]. Multifactorial pathogenesis, a combination of vascular, genetic, anatomical, and immune factors, has been implicated [1]. Risk factors include age, frailty [5], gender, myopia, genetics, family history, smoking, race, systemic hypotension and hypertension, vasospasm, use of systemic or topical steroids, migraine, obstructive sleep apnea syndrome [6], mitochondrial dysfunction [7,8,9], and most significantly, increased intraocular pressure (IOP).

It has been hypothesized that in some patients, glaucoma should be considered a neurodegenerative disease that originates in the brain but manifests as an eye disease, thus supporting the rising evidence of the existence of the brain–eye axis [4]. This has also been confirmed by increasing evidence showing retinal damage following retrograde trans-synaptic degeneration due to surgical or neurodegenerative processes [10,11,12].

The association between Alzheimer’s disease (AD) and glaucoma has also been investigated through various epidemiological studies with contrasting results [13,14]. While some studies suggest an increased prevalence of glaucoma in patients with dementia [12,15,16,17,18,19,20], other studies do not support this hypothesis [21,22,23,24]. In this regard, animal models and clinical studies have been elaborated to evaluate the relationship between AD and glaucoma [25,26,27,28,29,30,31,32,33,34,35]. In rat models, amyloid beta (Aβ) seems to be constitutively expressed by retinal ganglion cells (RGCs) when exposed to light [28]. On the other hand, Aβ seems to significantly overexpress under conditions of ocular hypertension, colocalizing with apoptotic RGCs. This suggests a role for Aβ in glaucomatous RGC death [29], emphasizing the need to identify new therapeutic strategies based on neuroprotection [9,36].

This paper aims to report the most relevant clinical findings, assessed using magnetic resonance imaging (MRI), that support the presence of neurodegenerative alterations in glaucoma and that suggest a connection between glaucoma and AD. It also aims to analyze possible common clinical biomarkers among these diseases. 

## 2. MRI and Clinical Biomarkers in Glaucoma

The involvement of the central nervous system (CNS) in glaucoma has been previously documented histologically, showing a greater loss of magnocellular tissue at the level of the lateral geniculate nucleus (LGN) in glaucomatous patients [37]. In another ex vivo study, Nissl-stained sections showed shrunken LGN. Concomitantly, MRI displayed high signal intensities in the LGN and a reduction in its size in glaucoma compared to controls. This was the first clinicopathological case of human glaucoma demonstrating brain degenerative changes involving the intracranial optic nerve, LGN, and visual cortex [38]. A subsequent in vivo study using a 1.5-Tesla MRI displayed LGN degeneration in human glaucoma, consistent with ex vivo primate and human neuropathological reports [39]. White matter has emerged as a potential biomarker through noninvasive in vivo imaging techniques. In particular, 3T MR imaging-derived in vivo parameters of the optic nerve and optic radiation have been shown to correlate with disease severity [40]. Subsequently, a correlation between the grade of atrophy in the LGN and disease severity has also been demonstrated using 3T-MRI [41]. These two early imaging studies performed noninvasively in vivo suggest the power of a possible new glaucoma imaging biomarker and simultaneously pave the way for numerous subsequent studies that have consistently confirmed these hypotheses. 

Neuroimaging techniques such as diffusion tensor imaging (DTI) and functional MRI (fMRI) have been used to evaluate the microstructural integrity of white-matter fibers and the functional activity of gray matter in vivo in the human brain. These imaging techniques elaborate on connectional models of brain function based on the diffusivity of water molecules along axons using mean diffusivity (MD) and fractional anisotropy (FA) [40,42].In previous studies, the optic radiation and optic nerves of patients with glaucoma showed significantly higher MD values and significantly lower FA values compared to healthy controls. The mean MD values for the optic nerves and the glaucoma stages varied consistently. on the contrary a negative correlation emerged between the mean FA for the optic nerves and the glaucoma stages. Thus, showing the usefulness of these parameters as complementary indicators of disease severity [40]. Furthermore, DTI parameters extrapolated from the optic nerve showed a good correlation with the morphological features of the optic nerve head and retinal nerve fiber layer (RNFL) thickness, which were documented using ophthalmological diagnostic imaging such as GDx-VCC, HRT-III, and OCT [43].

The analysis of MD and FA at the proximal level (to the optic nerve head) and distal level (at the orbital apex) using DTI was extremely useful for understanding the potential evolution of glaucoma. In early glaucoma, MD significantly increased at the proximal level, while in severe stages, both the proximal and the distal portions of the optic nerves showed pathological MD values. The extension of the disease to the entire visual pathway was also confirmed through the detection of significantly altered values of MD and FA at the level of the optic radiation [44].

However, optic nerve alterations not only originate from the eyes but also may be the result of central neurodegenerative processes [5,10,11,12,13,14,45].

The effect of brain damage, consequent to the surgical excision of an arteriovenous malformation in the right cerebral occipital lobe, on RGCs by trans-synaptic retrograde degeneration (TRD) has been documented by analyzing GCC and RNFL thickness. The study showed that retrograde neuronal degeneration can occur along the visual pathway, maintaining the topographic distribution of the GCC as projected to the visual cortex [10,11]. In this case, a neurodegenerative disease affecting the brain may trigger degenerative processes of the ocular structures. Consistently, it has been observed that there is a 27.5% frequency of glaucoma-like alterations among AD patients, which is five times higher than in healthy people. These data suggest that patients with AD may have a clinical picture that mimics glaucoma [12].

Diffusion kurtosis imaging (DKI) is an imaging technique that extends DTI and allows a more accurate description of the diffusion signal. When applied to white or gray matter, DKI indices can provide extended information compared to DTI [46].

Using both DTI and DKI, as well as whole-brain Tract-Based Spatial Statistics (TBSS) analysis, our group recently analyzed normal-appearing white matter (NAWM) changes in patients with primary open-angle glaucoma (POAG) as compared to healthy controls in a regionally unbiased, voxel-wise manner. Statistically significant differences in FA, kurtosis anisotropy (KA), mean kurtosis (MK), and radial kurtosis (RK) were found. TBSS showed lower FA values in the glaucoma group along and beyond the visual pathway. These data likely represent a loss of interconnection fibers caused by axonal damage covering the entire visual pathway and the superior cortices that are implicated in the integration of visual information [46].

The graph theoretical methods and a newly defined idea of subject-wise and group-wise disruption index allow us to analyze the topological properties of brain connectivity in patients affected by glaucoma. Multi-shell, multi-tissue tractography in conjunction with graph theory and the disruption index demonstrated a deep brain reorganization beyond the visual pathways. POAG patients showed a higher clustering coefficient in the calcarine gyrus, which contains the primary visual cortex (V1) and is an essential component of the visual pathway involved in vision-related function. Similar results were found in the lingual gyrus, which is implicated in topographical recognition, such as in environmental orientation. The lateral occipital gyrus also showed increased local efficiency and clustering coefficient. Being in the visual association area (V2), this region has a key role in the integration of visual information and conscious perceptions. The involvement of the lateral occipital area may explain why patients with glaucoma have impaired face recognition ability [47]. Statistically significant differences were also found in the paracentral lobe, which controls the motor and sensory innervation of the contralateral extremity. The higher clustering coefficient and local efficiency could therefore be interpreted as a more prominent role of motor control in this region, possibly in compensation for loss of sight. Higher values found in global strength, global efficiency, and global clustering coefficient in POAG may suggest a whole-brain structural reorganization in glaucoma, which is also supported by the differences found in the disruption index compared to controls. The higher disruption indices found in glaucoma point out the complementary role and significance of functional vs. structural connectivity in the analysis of subtle brain changes [47].

Resting-state functional magnetic resonance imaging (rs-fMRI) has been previously used to evaluate changes in functional brain connectivity in neurodegenerative diseases such as AD. In patients with glaucoma, graph-theoretical measures of integration, segregation, and centrality found statistically significant group-wise differences in subject-wise disruption indexes in all local metrics. The glaucoma group showed a lower disruption index k for all statistically significant comparisons, thus highlighting a complex functional brain network reorganization pattern. In particular, the left lobule VIIB of the cerebellar hemisphere was classified as a betweenness centrality hub in healthy controls but not in glaucoma patients. The right inferior occipital cortex behaved oppositely. Furthermore, the right angular gyrus was categorized as a spectral measure of the centrality hub in healthy controls but not in glaucoma patients. On the contrary, the right inferior temporal gyrus behaved oppositely. Lastly, the left lobule IX of the cerebellar hemisphere was classified as a local efficiency hub in glaucoma patients but not in healthy controls [48].

Interestingly, a study compared POAG patients and healthy controls by combining multi-shell diffusion-weighted imaging, multi-shell, multi-tissue probabilistic tractography, graph theoretical measures, and the disruption index. The study also evaluated the associations between the whole-brain structural connectivity measures and ophthalmological indices such as the visual field index (VFI) and two Optical Coherence Tomography (OCT) parameters, namely the macular Ganglion Cell Layer (GCL) and RNFL thicknesses. Global and local structural connectivity differences between POAG patients and controls extended beyond the primary visual pathway. They were especially localized in the left calcarine gyrus, left lateral occipital cortex, right lingual gyrus, and right paracentral lobule. Group-wise and subject-wise disruption indices also differed among the groups. Interestingly, RNFL thickness was negatively associated with local measures in the right amygdala, right inferior temporal gyrus, and right temporal pole. This data clearly showed that structural reorganization involves a variety of brain regions implicated in visual processing, motor control, and emotional/cognitive functions, identifying a pattern of brain structural changes correlated to POAG clinical severity [49].

In another study, ROC analysis confirmed that disruption indices were able to discriminate glaucoma patients from healthy controls with high sensitivity and specificity. Thus suggesting they were possibly good biomarkers for monitoring brain involvement and reorganization in glaucoma [50].

The alteration of the interconnection fibers may be the basis of the mismatch between clinical symptoms and visual field defects that are typical of glaucomatous patients and more evident in early disease stages. In this context, the impairment of peripheral vision only partially explains the difficulty these patients have performing actions that require a visual task (both simple and complex) [46]. Since peripheral visual field loss alone does not completely explain the disability of glaucomatous patients in carrying out daily living actions, the brain structural changes at the level of the areas implied in processing visual information could justify the discrepancy between signs and symptoms and underlie the analogy of this disease with neurodegenerative dementias and with the complex group of pathologies commonly referred to as “disconnection syndromes” [51].

## 3. MRI and Clinical Biomarkers in Alzheimer’s Disease

AD is among the most widespread neurodegenerative dementia diseases. It is characterized by the progressive and nonreversible loss of brain function, which adversely affects: (a) memory, (b) thinking, (c) language, (d) judgment, and (e) behavioral skills. Consequently, it affects the patient’s personality and social life [52,53].

Several longitudinal studies have analyzed clinical symptoms and investigated molecular, functional, and neuroimaging findings in AD patients, looking for early markers in the prodromal-asymptomatic phase.

AD involves cortical atrophy, which is adequately detected by computerized tomography (CT). However, MRI, which does not use ionizing radiation, is more sensitive to volumetric changes and can be used to rule out other causes of dementia. Several neuroimaging techniques using MRI are also available that are suitable for neurological evaluation, such as morphological, functional, and structural MRI sequences.

Using MRI sequences, (a) microstructural changes in AD associated with regional tau loading and local tissue atrophy, (b) ischemic and hypercellular lesions, (c) hemorrhage, (d) edema, (e) white matter changes predominantly in the frontal lobe, and (f) demyelination and axonal loss can be studied [54,55,56].

Using volumetric magnetic resonance imaging, it is possible to study the relationship between atrophic changes and the loss of neurons, which is well established and correlates well with clinical variability [57]. Cortical atrophy in AD is caused by the loss of neurons usually detectable in the medial and parietal temporal lobes, which spreads to the limbic gray matter structures and finally to the frontal cortical regions [55,56,58].

Chandra et al. [55] showed that the progression of atrophy follows Braak’s staging and is observed first in medial temporal lobe structures, including the entorhinal cortex and hippocampus. Hippocampal volumes in AD patients are reduced compared to controls. The presence of diffuse hippocampal atrophy in AD patients correlates with deficits in executive functioning and memory. As the disease progresses, the atrophy extends to the rest of the medial temporal lobe, where gray matter loss occurs in the medial temporal gyrus, the parahippocampal and fusiform gyri, and the temporal pole. However, the Blennow et al. [58] study points out that atrophy of the hippocampus and entorhinal cortex is also found in other types of dementia, such as frontotemporal and vascular dementia. The results show that hippocampal atrophy can differentiate AD patients from the group of normal elderly controls with an accuracy in the range of 80–90%.

In the study by Belathur et al. [59], it is reported that cortical thickness can correctly classify AD patients and healthy subjects with 90% accuracy, 96% sensitivity, and 76% specificity. 

Leocadi et al. [60] reported that hippocampal volume is considered an appropriate marker of AD progression and appears suitable for monitoring treatment efficacy. 

Medial temporal lobe atrophy correlates with memory decline in patients with mild cognitive impairment (MCI).

It must be emphasized that brain atrophy is not a specific sign in AD patients. The results of the study by Mueller et al. [57] showed: (a) substantial differences in the rate of brain atrophy in the healthy brain and the brain affected by neurodegeneration; (b) an increasing tendency of limbic and temporal lobe atrophy in AD patients, while normal aging affects the frontal and parietal gray matter. Miller-Thomas et al. [61] reported in their study that the hippocampus shows more extensive neuronal loss among brain volumes in AD patients than in controls. In the study by Qian et al. [62], the cognitive impairment observed in patients with MCI was linked to hippocampal atrophy; however, the change in thalamus volume was correlated with deficits in language, executive, and visuospatial abilities. Thalamic volume atrophy has been observed as an early sign of MCI and AD.

fMRI is commonly employed to study changes in functional brain connectivity in a vast number of conditions, including neurodegenerative diseases such as Parkinson’s or Alzheimer’s disease. The interest in the so-called functional connectome (i.e., the complex network of cross-talks between brain areas) is ever-increasing [63,64,65,66].

Both the studies by Chandra et al. [55] and Sperling et al. [67] on episodic memory in AD reported decreased activation in the hippocampal, parahippocampal, and medial temporal structures during memorization. Furthermore, the Chandra et al. [55] study demonstrated impairments in working memory, visuospatial ability, attention, semantic cognition, and motor performance in AD, compared with MCI, in which more dominant changes were found in attention and working memory. In addition, Yamasaki et al. [68] revealed that many AD patients also have visual-spatial deficits, and Cheng et al. [69] examining rs-fMRI changes in AD found worse cognitive improvements in (a) the right rectus gyrus, (b) the right precentral gyrus, and (c) the left superior temporal gyrus. Leocadi et al. [60] explore early and late MCI, and task-based fMRI has provided insights into the most affected dysfunctions in sensorimotor networks that accompany disease worsening and could be potential biomarkers for progression from MCI to AD. While fMRI demonstrated impairments in working memory, visuospatial ability, attention, semantic cognition, and motor performance in AD, more dominant changes in attention and working memory were found in MCI [55].

Penalba-Sánchez et al. [70] reported that AD is identified at an early stage using functional connectivity (FC), i.e., the statistical dependence between two or more brain regions, through new analysis techniques. In this study, static and dynamic HR were assessed using different approaches. AD patients were evaluated by rs-fMRI. The blood-oxygen-level-dependent signals of 116 regions from 4 groups of participants, namely healthy controls (HC), early mild cognitive impairment (EMCI), late mild cognitive impairment (LMCI), and AD, were extracted and analyzed. Static and dynamic HR were extracted using Pearson correlation, sliding-windows correlation analysis, and point process analysis. In addition, graph-theoretic measures were calculated to explore network segregation and integration. The results showed a longer characteristic path length and a decrease in the degree of EMCI compared to the other groups. More specifically, AD presented an increased FC between the angular gyrus (left) and cerebellar crust II (left) compared to EMCI and between the superior occipital gyrus (right) and calcarine fissure (left). Compared with HC, AD also had increased FC between the thalamus and inferior frontal gyrus (left), between the middle temporal gyrus (left) and the caudate nucleus (left), between the vermis 9 and superior occipital (left), and between the caudate (right) and inferior frontal gyrus (left). The only regions exhibiting reduced FC in AD compared to HC were the middle temporal gyrus (right) and superior occipital gyrus (left).

Machine learning techniques applied to neuroimaging have encouraged the implementation of models to diagnose brain disorders such as Alzheimer’s disease in their early stages. Predicting the exact stage of AD is challenging; deep learning techniques try to evaluate it with higher precision. The study by Sarraf et al. [71] introduced an optimized architecture to predict group membership by separating healthy adults, mild cognitive impairment, and Alzheimer’s brains within the same age group (>75 years) via structural MRI and functional at rest. The model achieved F1 scores of 97% ± 0.0 and 99.55% ± 0.39 from the functional imaging modality test sets.

Zhang et al. [72] proposed a study on the classification of brain disorders in rs-fMRI via Local-to-Global Graph Neural Networks. Specifically, this technique aims to learn a region of interest graph neural networks based on a regional brain graph, analyze the characteristics of local brain regions, and identify biomarkers. The top 10 brain regions for AD classification, with the greatest weight, are mainly the hippocampus, superior parietal lobule, temporal gyrus, inferior frontal gyrus, insular cortex, etc., which have been reported as responsible for short-term memory and early stages of AD.

In the study by Chen et al. [73], 3T rs-fMRI data were obtained from healthy young controls (YC), healthy elderly controls (SC), and AD patients. Fractional amplitude of low-frequency fluctuations (fALFF), regional homogeneity (ReHo), and degree centrality (DC) were analyzed. Group differences in agreement were compared globally, within seven intrinsic brain networks, and on a voxel-by-voxel basis. Overall agreement was lowest in AD among the three groups, with similar differences for individual parameters. When comparing AD with SC, reductions in concordance were found in each of the networks studied, except the limbic network. A lower overall agreement was observed for SC versus YC, with no network-level difference. Voxel analyses revealed lower agreement in the right mid-temporal gyrus in AD versus SC and lower agreement in the left mid-frontal gyrus in SC versus YC. Lower fALFF was observed in the right angular gyrus in AD compared to SC, but ReHo and DC showed no group differences. Finally, matching resting-state measures differentiate AD from healthy aging and may represent a novel imaging marker in AD.

Diffusion MRI techniques are appropriate for estimating damage to structural neurological integrity caused by neurodegeneration, acute ischemia, tumors, and other lesions, edemas, infections, and protein plaques [74,75,76,77,78,79].

Studies of white matter connectivity in patients with various cognitive impairments have shown worse overall network density, reduced nodal strength, and lower white matter fiber tract integrity associated with lower memory performance [60,76]. These mirror changes in synaptoplasticity, which is likely disturbed simultaneously with disease progression. Two hypotheses have been proposed to explain white matter changes in AD patients: (a) Wallerian degeneration altering white matter microstructure or (b) diffuse demyelination of affected tracts [76]. Impairments in diffusivity and FA have also been linked to memory deficits and executive dysfunction [55]. Reduced FA and higher tissue diffusivity in AD compared to controls have been reported in the frontal and temporal lobes, posterior cingulate, corpus callosum, superior longitudinal, and uncinate bundles [76]. However, a discriminative model for AD suggested lower AF in the parahippocampal cingulate, crus, and body of the fornix [80].

Interestingly, increased diffusivity occurs in the parietal and temporal lobes of MCI and AD patients; however, changes in the frontal and occipital regions have been found in AD [55]. Recently, it has been suggested that functional brain connectivity, especially in the frontolimbic circuit, may predict the presence of neuropsychiatric symptoms, including AD progression [55,60]. The alteration in diffusivity could also distinguish AD from other dementias. A lower FA was certainly found in the frontal areas of patients with frontotemporal dementia, while this was not observed in patients with AD. However, greater diffusivity was found in the parietal and temporal regions in patients with AD than in those with frontotemporal dementia [55].

Studies with biophysical multi-compartment models [81], such as the Neurite Orientation Dispersion and Density Imaging (NODDI) model, have shown changes in the entorhinal, inferior temporal, temporal media, fusiform, and precuneus cortices of AD patients compared with controls. Additionally, these changes were also seen in the precentral gyrus (which is connected to the primary motor cortex). Moreover, the white matter bundles of the parieto-occipital lobes have shown correlations with visuo-spatial and visuo-perceptual cognitive performance in AD. Furthermore, measurements of WM changes are important for mechanistic understanding of the multifactorial pathways by which AD causes cognitive dysfunction [82,83,84]. Diffusion MRI may allow microscopic characterization of white matter degeneration in the early stages of Alzheimer’s disease because: (a) mild cognitive decline results in microstructural changes in white matter tracts; (b) changes include increased axonal dispersion and decreased tissue restriction; (c) diffusion metrics are associated with cognitive outcomes in AD patients [85].

## 4. Common Biomarkers in Glaucoma and Alzheimer’s Disease Diagnosis

The pathophysiological process underlying AD is in many aspects similar to that of glaucoma. Numerous epidemiological studies using immunohistochemical and animal data support the link between the two diseases [86].

Since glaucoma involves other brain areas than the central visual ones (for example, the temporal lobes) that are superimposable to the areas involved in AD patients, this suggests a possible link between the two pathologies. However, the link between AD and glaucoma is not yet fully clear and explicit, and there are still contrasting opinions [87,88].

The study by Hanafiah et al. [89] evaluated MRI findings suggestive of Alzheimer’s disease in patients with primary open-angle glaucoma using morphological analysis of a single sequence (the 3D spoiled T1 fast gradient echo). The existence of medial temporal atrophy and parietal lobe atrophy in glaucoma patients compared to control subjects was assessed using the medial temporal atrophy (MTA) and posterior cortical atrophy (PCA) scoring systems using the MRI sequence. The results showed that there was a statistically significant difference between the PCA score in glaucoma patients and the control group; in contrast, there was no statistically significant difference between the MTA score between the two groups. The study shows that the PCA score method used in AD patients correlates with glaucoma patients. Therefore, the PCA score method can be used in the diagnosis and monitoring of both AD and glaucoma patients. Measuring parietal lobe atrophy using this rating scale adds value to discriminating AD from controls and other dementias using MRI [24]. Some articles report that there is no correlation between AD and glaucoma [21,23] or, if there is a correlation, it may be due to chance [90,91]. Therefore, it is too much to assume a direct association from the Hanafiah et al. [89] study alone. 

In the study by Diaz-Torres et al. [92], the genetic and causal relationship between glaucoma patients and neurodegenerative disorders (such as those of AD) was evaluated by exploiting genome association data obtained from MRI studies of the brain. The study revealed a genetic overlap and a causal relationship between patients with glaucoma and its related phenotypes (for example, intraocular pressure and optic nerve morphological traits) and brain morphology in 19 regions. Furthermore, 11 loci emerged with a significant local genetic correlation and a high probability of sharing the same causal variant between neurodegenerative disorders and glaucoma. Interestingly, a region on chromosome 17 corresponding to the microtubule-associated protein tau, a known risk locus for Alzheimer’s disease, was shared among patients with glaucoma, optic nerve degeneration traits, and Alzheimer’s. Despite these local genetic overlaps, the study found no clear evidence of a causal association between these neurodegenerative disorders and glaucoma.

The article by Bogolepova et al. [93] presented data on biomarkers for early diagnosis of patients with AD. Neuroimaging (through MRI morphological sequences) and ophthalmological (through optical coherence tomography) markers are studied. The article shows the relationship between AD and glaucoma and considers a case of AD in a patient with primary open-angle glaucoma.

Further research, especially at the molecular, genetic, and imaging levels, is needed to investigate whether there are direct links between the two diseases (AD and glaucoma). However, more studies are needed to further investigate the association between the two diseases and the use of brain MRI. In fact, the various studies reported in the literature that use biomarkers obtained through MRI imaging have limitations above all because patients affected by AD and glaucoma are not studied contextually with MRI but only on clinical scales.

## 5. Future Directions

The identification of clinical and neuroimaging biomarkers for the detection and diagnosis of glaucoma and AD is very important regarding structural, functional, and molecular brain changes underlying the observed clinical symptoms. 

Studies with various MRI techniques have demonstrated patterns that allow the study of brain atrophy in various neurodegenerative disorders, which may be useful in differential diagnosis. Advanced MRI sequences, such as diffusion-based imaging and functional MRI, provide important insights into underlying biological changes in pathology and for the development of new clinical indications. Finally, advances in imaging enable the multidisciplinary team to study and follow up on the two different pathologies.

Combining the various neuroimaging techniques may be useful, if not essential, for the differential diagnosis of the two symptoms. Further studies with advanced magnetic resonance imaging techniques applied to both glaucoma and AD can provide a complete picture of the pathology associated with the neurodegenerative disease. In addition, neuroimaging techniques may be useful in combination clinical trials so that overlaps and/or differences between glaucoma and AD can be highlighted to monitor disease-related changes and be used as endpoints to complement current clinical outcome measures.

Although the precise pathological mechanisms of glaucoma and AD are still an enigma, the multidisciplinary group can evaluate through neuroimaging studies the pathogenesis of these diseases by directly comparing neuroimaging outcomes. 

Considering the growing and pressing need to identify biomarkers that can predict the early development of glaucoma or AD and to track both disease progression and efficacy of treatments in clinical trials, it is necessary to pool all possible knowledge in terms of data and technological resources in order to speed the generation of new effective non-invasive biomarkers. 

In this context, promising results seem to come from the use of artificial intelligence (AI) and, in particular, from machine learning models such as those tested from retinal imaging datasets [93] (retinal photographs) for the diagnosis of AD, as recently described in the study of Cheung and colleagues.

This study and similar ones suggest that AI will have an increasingly growing role in ophthalmology, radiology, and neurology for the identification of biomarkers that meet the aforementioned needs. 

Moreover, this could lead to a better understanding of the diseases, and in a clinical context, these new promising methods may even be able to diagnose glaucoma in borderline cases, avoiding delays in starting treatments or, in a translational research context, speeding drug discovery and development as well as the expensive process of developing novel drugs. 

Sharing all available knowledge, including the most technologically advanced, will be necessary to bridge the gap between preclinical and clinical studies and to understand the biological mechanisms shared by the two diseases. It will also be necessary to test new molecules to identify targeted therapies and ultimately progress toward personalized approaches for complex conditions, such as those occurring in the neurodegenerative process of glaucoma and AD.

## References

[1] Allison K., Patel D., Alabi O. (2020). Epidemiology of Glaucoma: The Past, Present, and Predictions for the Future. Cureus.

[2] Cedrone C., Mancino R., Cerulli A., Cesareo M., Nucci C. (2008). Epidemiology of primary glaucoma: Prevalence, incidence, and blinding effects. Prog. Brain Res..

[3] Cedrone C., Mancino R., Ricci F., Cerulli A., Culasso F., Nucci C. (2012). The 12-year Incidence of Glaucoma and Glaucoma-related Visual Field Loss in Italy. J. Glaucoma.

[4] Martucci A., Nucci C., Pinazo-Duran M.D. (2023). Editorial: New perspectives in glaucoma pathophysiology, diagnosis, and treatment. Front. Med..

[5] Nucci C., Martucci A., Martorana A., Sancesario G.M., Cerulli L. (2011). Glaucoma progression associated with altered cerebral spinal fluid levels of amyloid beta and tau proteins. Clin. Exp. Ophthalmol..

[6] Cesareo M., Giannini C., Martucci A., Di Marino M., Pocobelli G., Aiello F., Mancino R., Nucci C. (2020). Links between obstructive sleep apnea and glaucoma neurodegeneration. Prog. Brain Res..

[7] Nucci C., Martucci A., Mancino R., Cerulli L. (2013). Glaucoma progression associated with Leber’s hereditary optic neuropathy. Int. Ophthalmol..

[8] Martucci A., Nucci C. (2019). Evidence on neuroprotective properties of coenzyme Q10 in the treatment of glaucoma. Neural Regen. Res..

[9] Martucci A., Reurean-Pintilei D., Manole A. (2019). Bioavailability and sustained plasma concentrations of CoQ10 in healthy volunteers by a novel oral timed-release preparation. Nutrients.

[10] Martucci A., Cesareo M., Nucci C., Mancino R. (2018). Macular ganglion cells alteration in a patient with left homonymous hemianopia subsequent to surgical excision of an arteriovenous malformation. Am. J. Ophthalmol. Case Rep..

[11] Mancino R., Cesareo M., Martucci A., Carlo E.D., Ciuffoletti E., Giannini C., Morrone L.A., Nucci C., Garaci F. (2019). Neurodegenerative process linking the eye and the brain. Curr. Med. Chem..

[12] Cesareo M., Martucci A., Ciuffoletti E., Mancino R., Cerulli A., Sorge R.P., Martorana A., Sancesario G., Nucci C. (2015). Association between Alzheimer’s disease and glaucoma: A study based on heidelberg retinal tomography and frequency doubling technology perimetry. Front. Neurosci..

[13] Mancino R., Martucci A., Cesareo M., Giannini C., Corasaniti M.T., Bagetta G., Nucci C. (2018). Glaucoma and Alzheimer disease: One age-related neurodegenerative disease of the brain. Curr. Neuropharmacol..

[14] Nucci C., Martucci A., Cesareo M., Garaci F., Morrone L.A., Russo R., Corasaniti M.T., Bagetta G., Mancino R., Corasaniti M.T. (2015). Links among glaucoma, neurodegenerative, and vascular diseases of the central nervous systemb. Prog. Brain Res..

[15] Chandra V., Bharucha N.E., Schoenberg B.S. (1986). Conditions associated with Alzheimer’s disease at death: Case-control study. Neurology.

[16] Bayer A.U., Ferrari F., Erb C. (2002). High Occurrence Rate of Glaucoma among Patients with Alzheimer’s Disease. Eur. Neurol..

[17] Bayer A.U., Keller O.N., Ferrari F., Maag K.-P. (2002). Association of glaucoma with neurodegenerative diseases with apoptotic cell death: Alzheimer’s disease and Parkinson’s disease. Am. J. Ophthalmol..

[18] Tamura H., Kawakami H., Kanamoto T., Kato T., Yokoyama T., Sasaki K., Izumi Y., Matsumoto M., Mishima H.K. (2006). High frequency of open-angle glaucoma in Japanese patients with Alzheimer’s disease. J. Neurol. Sci..

[19] Lin I.C., Wang Y.H., Wang T.J., Wang I.J., Shen Y., Chi N.F., Chien L.N. (2014). Glaucoma, Alzheimer’s disease, and Parkinson’s disease: An 8-year population-based follow-up study. PLoS ONE.

[20] Kurna S.A., Akar G., Altun A., Agirman Y., Gozke E., Sengor T. (2014). Confocal scanning laser tomography of the optic nerve head on the patients with Alzheimer’s disease compared to glaucoma and control. Int. Ophthalmol..

[21] Kessing L.V., Lopez A.G., Andersen P.K., Kessing S.V. (2007). No Increased Risk of Developing Alzheimer Disease in Patients With Glaucoma. J. Glaucoma.

[22] Bach-Holm D., Kessing S.V., Mogensen U., Forman J.L., Andersen P.K., Kessing L.V. (2012). Normal tension glaucoma and Alzheimer disease: Comorbidity?. Acta Ophthalmol..

[23] Keenan T.D.L., Goldacre R., Goldacre M.J. (2015). Associations between primary open angle glaucoma, Alzheimer’s disease and vascular dementia: Record linkage study. Br. J. Ophthalmol..

[24] Ou Y., Grossman D.S., Lee P.P., Sloan F.A. (2012). Glaucoma, Alzheimer Disease and Other Dementia: A Longitudinal Analysis. Ophthalmic Epidemiol..

[25] Della Santina L., Inman D.M., Lupien C.B., Horner P.J., Wong R.O.L. (2013). Differential Progression of Structural and Functional Alterations in Distinct Retinal Ganglion Cell Types in a Mouse Model of Glaucoma. J. Neurosci..

[26] Jakobs T.C., Libby R.T., Ben Y., John S.W.M., Masland R.H. (2005). Retinal ganglion cell degeneration is topological but not cell type specific in DBA/2J mice. J. Cell Biol..

[27] Poon W.W., Blurton-Jones M., Tu C.H., Feinberg L.M., Chabrier M.A., Harris J.W., Jeon N.L., Cotman C.W. (2011). β-Amyloid impairs axonal BDNF retrograde trafficking. Neurobiol. Aging.

[28] Wang J., Zhu C., Xu Y., Liu B., Wang M., Wu K. (2011). Development and Expression of Amyloid-β Peptide 42 in Retinal Ganglion Cells in Rats. Anat. Rec. Adv. Integr. Anat. Evol. Biol..

[29] Guo L., Salt T.E., Luong V., Wood N., Cheung W., Maass A., Ferrari G., Russo-Marie F., Sillito A.M., Cheetham M.E. (2007). Targeting amyloid-β in glaucoma treatment. Proc. Natl. Acad. Sci. USA.

[30] Kipfer-Kauer A., McKinnon S.J., Frueh B.E., Goldblum D. (2010). Distribution of Amyloid Precursor Protein and Amyloid-β in Ocular Hypertensive C57BL/6 Mouse Eyes. Curr. Eye Res..

[31] McKinnon S.J., Lehman D.M., Kerrigan-Baumrind L.A., Merges C.A., Pease M.E., Kerrigan D.F., Ransom N.L., Tahzib G.N., Reitsamer H.A., Levkovithc-Verbin H. (2002). Caspase activation and amyloid precursor protein cleavage in rat ocular hypertension. Investig. Ophthalmol. Vis. Sci..

[32] Gupta V.K., Chitranshi N., Gupta V.B., Golzan M., Dheer Y., Vander Wall R., Georgevsky D., King A.E., Vickers J.C., Chung R. (2016). Amyloid β accumulation and inner retinal degenerative changes in Alzheimer’s disease transgenic mouse. Neurosci. Lett..

[33] Perez S.E., Lumayag S., Kovacs B., Mufson E.J., Xu S. (2009). β-Amyloid Deposition and Functional Impairment in the Retina of the APPswe/PS1ΔE9 Transgenic Mouse Model of Alzheimer’s Disease. Investig. Opthalmol. Vis. Sci..

[34] Gasparini L., Anthony Crowther R., Martin K.R., Berg N., Coleman M., Goedert M., Spillantini M.G. (2011). Tau inclusions in retinal ganglion cells of human P301S tau transgenic mice: Effects on axonal viability. Neurobiol. Aging.

[35] Martucci A., Picchi E., Di Giuliano F., Pocobelli G., Mancino R., Toschi N., Russo R., Floris R., Garaci F., Nucci C. (2022). Imaging biomarkers for Alzheimer’s disease and glaucoma: Current and future practices. Curr. Opin. Pharmacol..

[36] Nucci C., Russo R., Martucci A., Giannini C., Garaci F., Floris R., Bagetta G., Morrone L.A. (2016). New strategies for neuroprotection in glaucoma, a disease that affects the central nervous system. Eur. J. Pharmacol..

[37] Chaturvedi N., Hedley-Whyte E.T., Dreyer E.B. (1993). Lateral geniculate nucleus in glaucoma. Am. J. Ophthalmol..

[38] Gupta N. (2006). Human glaucoma and neural degeneration in intracranial optic nerve, lateral geniculate nucleus, and visual cortex. Br. J. Ophthalmol..

[39] Gupta N., Greenberg G., de Tilly L.N., Gray B., Polemidiotis M., Yucel Y.H. (2009). Atrophy of the lateral geniculate nucleus in human glaucoma detected by magnetic resonance imaging. Br. J. Ophthalmol..

[40] Garaci F.G., Cozzolino V., Nucci C., Gaudiello F., Ludovici A., Lupattelli T., Floris R., Simonetti G. (2008). Advances in neuroimaging of the visual pathways and their use in glaucoma. Prog. Brain Res..

[41] Garaci F.G., Bolacchi F., Cerulli A., Melis M., Spanò A., Cedrone C., Floris R., Simonetti G., Nucci C. (2009). Optic Nerve and Optic Radiation Neurodegeneration in Patients with Glaucoma: In Vivo Analysis with 3-T Diffusion-Tensor MR Imaging. Radiology.

[42] Nucci C., Mancino R., Martucci A., Bolacchi F., Manenti G., Cedrone C., Culasso F., Floris R., Cerulli L., Garaci F.G. (2012). 3-T Diffusion tensor imaging of the optic nerve in subjects with glaucoma: Correlation with GDx-VCC, HRT-III and Stratus optical coherence tomography findings. Br. J. Ophthalmol..

[43] Bolacchi F., Garaci F.G., Martucci A., Meschini A., Fornari M., Marziali S., Mancino R., Squillaci E., Floris R., Cerulli L. (2012). Differences between proximal versus distal intraorbital optic nerve diffusion tensor magnetic resonance imaging properties in glaucoma patients. Investig. Ophthalmol. Vis. Sci..

[44] Nucci C., Martucci A., Cesareo M., Mancino R., Russo R., Bagetta G., Cerulli L., Garaci F.G. (2013). Brain involvement in glaucoma: Advanced neuroimaging for understanding and monitoring a new target for therapy. Curr. Opin. Pharmacol..

[45] Nucci C., Garaci F., Altobelli S., Di Ciò F., Martucci A., Aiello F., Lanzafame S., Di Giuliano F., Picchi E., Minosse S. (2020). Diffusional kurtosis imaging of white matter degeneration in glaucoma. J. Clin. Med..

[46] Di Cio F., Garaci F., Minosse S., Passamonti L., Martucci A., Lanzafame S., Di Giuliano F., Picchi E., Mancino R., Guerrisi M. Disruption of structural brain networks in Primary Open Angle Glaucoma. Proceedings of the Annual International Conference of the IEEE Engineering in Medicine and Biology Society, EMBS.

[47] Minosse S., Garaci F., Martucci A., Lanzafame S., Di Giuliano F., Picchi E., Cesareo M., Mancino R., Guerrisi M., Floris R. Disruption of brain network organization in primary open angle glaucoma. Proceedings of the Annual International Conference of the IEEE Engineering in Medicine and Biology Society, EMBS.

[48] Di Ciò F., Garaci F., Minosse S., Passamonti L., Martucci A., Lanzafame S., Di Giuliano F., Picchi E., Cesareo M., Guerrisi M.G. (2020). Reorganization of the structural connectome in primary open angle Glaucoma. Neuroimage Clin..

[49] Minosse S., Garaci F., Martucci A., Lanzafame S., Giuliano F.D., Picchi E., Cesareo M., Mancino R., Guerrisi M., Pistolese C.A. (2019). Primary open angle glaucoma is associated with functional brain network reorganization. Front. Neurol..

[50] Martucci A., Cesareo M., Toschi N., Garaci F., Bagetta G., Nucci C. (2020). Brain networks reorganization and functional disability in glaucoma. Prog. Brain Res..

[51] Beshir S.A., Aadithsoorya A.M., Parveen A., Goh S.S.L., Hussain N., Menon V.B. (2022). Aducanumab Therapy to Treat Alzheimer’s Disease: A Narrative Review. Int. J. Alzheimers Dis..

[52] Michailidis M., Moraitou D., Tata D.A., Kalinderi K., Papamitsou T., Papaliagkas V. (2022). Alzheimer’s Disease as Type 3 Diabetes: Common Pathophysiological Mechanisms between Alzheimer’s Disease and Type 2 Diabetes. Int. J. Mol. Sci..

[53] Berlow Y.A., Wells W.M., Ellison J.M., Sung Y.H., Renshaw P.F., Harper D.G. (2010). Neuropsychiatric correlates of white matter hyperintensities in Alzheimer’s disease. Int. J. Geriatr. Psychiatry.

[54] Chandra A., Dervenoulas G., Politis M. (2019). Magnetic resonance imaging in Alzheimer’s disease and mild cognitive impairment. J. Neurol..

[55] Patel K.P., Wymer D.T., Bhatia V.K., Duara R., Rajadhyaksha C.D. (2020). Multimodality imaging of dementia: Clinical importance and role of integrated anatomic and molecular imaging. Radiographics.

[56] Mueller S.G., Schuff N., Weiner M.W. (2006). Evaluation of treatment effects in Alzheimer’s and other neurodegenerative diseases by MRI and MRS. NMR Biomed..

[57] Blennow K., de Leon M.J., Zetterberg H. (2006). Alzheimer’s disease. Lancet.

[58] Belathur Suresh M., Fischl B., Salat D.H. (2018). Factors influencing accuracy of cortical thickness in the diagnosis of Alzheimer’s disease. Hum. Brain Mapp..

[59] Leocadi M., Canu E., Calderaro D., Corbetta D., Filippi M., Agosta F. (2020). An update on magnetic resonance imaging markers in AD. Ther. Adv. Neurol. Disord..

[60] Miller-Thomas M.M., Sipe A.L., Benzinger T.L.S., McConathy J., Connolly S., Schwetye K.E. (2016). Multimodality review of amyloid-related diseases of the central nervous system. Radiographics.

[61] Qian L., Liu R., Qin R., Zhao H., Xu Y. (2018). The associated volumes of sub-cortical structures and cognitive domain in patients of Mild Cognitive Impairment. J. Clin. Neurosci..

[62] Bullmore E., Sporns O. (2009). Complex brain networks: Graph theoretical analysis of structural and functional systems. Nat. Rev Neurosci..

[63] Sporns O., Betzel R.F. (2016). Modular Brain Networks. Annu. Rev. Psychol..

[64] Sporns O. (2013). The human connectome: Origins and challenges. Neuroimage.

[65] Minosse S., Garaci F., Martino F., Di Mauro R., Melis M., Di Giuliano F., Picchi E., Floris R., Guerrisi M., Di Girolamo S. Global and local reorganization of brain network connectivity in sudden sensorineural hearing loss. Proceedings of the Annual International Conference of the IEEE Engineering in Medicine and Biology Society, EMBS.

[66] Sperling R. (2011). The potential of functional MRI as a biomarker in early Alzheimer’s disease. Neurobiol. Aging.

[67] Yamasaki T., Muranaka H., Kaseda Y., Mimori Y., Tobimatsu S. (2012). Understanding the pathophysiology of Alzheimer’s disease and mild cognitive impairment: A mini review on fMRI and ERP studies. Neurol. Res. Int..

[68] Cheng J., Yang H., Zhang J. (2019). Donepezil’s effects on brain functions of patients with alzheimer disease: A regional homogeneity study based on resting-state functional magnetic resonance imaging. Clin. Neuropharmacol..

[69] Penalba-Sánchez L., Oliveira-Silva P., Sumich A.L., Cifre I. (2023). Increased functional connectivity patterns in mild Alzheimer’s disease: A rsfMRI study. Front. Aging Neurosci..

[70] Sarraf S., Sarraf A., DeSouza D.D., Anderson J.A.E., Kabia M. (2023). OViTAD: Optimized Vision Transformer to Predict Various Stages of Alzheimer’s Disease Using Resting-State fMRI and Structural MRI Data. Brain Sci..

[71] Zhang H., Song R., Wang L., Zhang L., Wang D., Wang C., Wang C., Zhang W. (2023). Classification of Brain Disorders in rs-fMRI via Local-to-Global Graph Neural Networks. IEEE Trans. Med. Imaging.

[72] Chen X., Onur O.A., Richter N., Fassbender R., Gramespacher H., Befahr Q., von Reutern B., Dillen K., Jacobs H.I.L., Kukolja J. (2021). Concordance of Intrinsic Brain Connectivity Measures Is Disrupted in Alzheimer’s Disease. Brain Connect..

[73] Drake-Pérez M., Boto J., Fitsiori A., Lovblad K., Vargas M.I. (2018). Clinical applications of diffusion weighted imaging in neuroradiology. Insights Imaging.

[74] Gaddamanugu S., Shafaat O., Sotoudeh H., Sarrami A.H., Rezaei A., Saadatpour Z., Singhal A. (2022). Clinical applications of diffusion-weighted sequence in brain imaging: Beyond stroke. Neuroradiology.

[75] Harrison J.R., Bhatia S., Tan Z.X., Mirza-Davies A., Benkert H., Tax C.M.W., Jones D.K. (2020). Imaging Alzheimer’s genetic risk using diffusion MRI: A systematic review. Neuroimage Clin..

[76] Minosse S., Picchi E., Di Giuliano F., Di Cio F., Pistolese C.A., Sarmati L., Teti E., Andreoni M., Floris R., Guerrisi M. Compartmental models for diffusion weighted MRI reveal widespread brain changes in HIV-infected patients. Proceedings of the Annual International Conference of the IEEE Engineering in Medicine and Biology Society, EMBS.

[77] Minosse S., Marzi S., Piludu F., Vidiri A. (2017). Correlation study between DKI and conventional DWI in brain and head and neck tumors. Magn. Reson. Imaging.

[78] Minosse S., Marzi S., Piludu F., Boellis A., Terrenato I., Pellini R., Covello R., Vidiri A. (2020). Diffusion kurtosis imaging in head and neck cancer: A correlation study with dynamic contrast enhanced MRI. Phys. Medica.

[79] Perea R.D., Rabin J.S., Fujiyoshi M.G., Neal T.E., Smith E.E., Van Dijk K.R.A., Hedden T. (2018). Connectome-derived diffusion characteristics of the fornix in Alzheimer’s disease. Neuroimage Clin..

[80] Martinez-Heras E., Grussu F., Prados F., Solana E., Llufriu S. (2021). Diffusion-Weighted Imaging: Recent Advances and Applications. Semin. Ultrasound CT MRI.

[81] Parker T.D., Slattery C.F., Zhang J., Nicholas J.M., Paterson R.W., Foulkes A.J.M., Malone I.B., Thomas D.L., Modat M., Cash D.M. (2018). Cortical microstructure in young onset Alzheimer’s disease using neurite orientation dispersion and density imaging. Hum. Brain Mapp..

[82] Slattery C.F., Zhang J., Paterson R.W., Foulkes A.J.M., Carton A., Macpherson K., Mancini L., Thomas D.L., Modat M., Toussaint N. (2017). ApoE influences regional white-matter axonal density loss in Alzheimer’s disease. Neurobiol. Aging.

[83] Tian J., Raghavan S., Reid R.I., Przybelski S.A., Lesnick T.G., Gebre R.K., Graff-Radford J., Schwarz C.G., Lowe V.J., Kantarci K. (2023). White Matter Degeneration Pathways Associated With Tau Deposition in Alzheimer Disease. Neurology.

[84] Wen Q., Mustafi S.M., Li J., Risacher S.L., Tallman E., Brown S.A., West J.D., Harezlak J., Farlow M.R., Unverzagt F.W. (2019). White matter alterations in early-stage Alzheimer’s disease: A tract-specific study. Alzheimer’s Dement. Diagn. Assess. Dis. Monit..

[85] Sen S., Saxena R., Tripathi M., Vibha D., Dhiman R. (2020). Neurodegeneration in Alzheimer’s disease and glaucoma: Overlaps and missing links. Eye.

[86] Ghiso J.A. (2013). Alzheimer’s disease and glaucoma: Mechanistic similarities and differences. J. Glaucoma.

[87] Sivak J.M. (2013). The aging eye: Common degenerative mechanisms between the Alzheimer’s brain and retinal disease. Investig Ophthalmol. Vis. Sci..

[88] Hanafiah M., Johari B., Ab Mumin N., Musa A.A., Hanafiah H. (2022). MRI findings suggestive of Alzheimer’s disease in patients with primary open angle glaucoma–a single sequence analysis using rapid 3D T1 spoiled gradient echo. Br. J. Radiol..

[89] Koedam E.L.G.E., Lehmann M., Van Der Flier W.M., Scheltens P., Pijnenburg Y.A.L., Fox N., Barkhof F., Wattjes M.P. (2011). Visual assessment of posterior atrophy development of a MRI rating scale. Eur. Radiol..

[90] Kuang G., Salowe R., O’Brien J. (2023). Genetic Factors Implicated in the Investigation of Possible Connections between Alzheimer’s Disease and Primary Open Angle Glaucoma. Genes.

[91] Diaz-Torres S., He W., Thorp J., Seddighi S., Mullany S., Hammond C.J., Hysi P.G., Pasquale L.R., Khawaja A.P., Hewitt A.W. (2023). Disentangling the genetic overlap and causal relationships between primary open-angle glaucoma, brain morphology and four major neurodegenerative disorders. EBioMedicine.

[92] Bogolepova A.N., Makhnovich E.V., Kovalenko E.A., Osinovskaya N.A., Beregov M.M., Zhuravleva A.N. (2023). Modern possibilities of early diagnosis of Alzheimer’s disease in patients with primary open-angle glaucoma. Zhurnal Nevrol. I Psikhiatrii Im SS Korsakova.

[93] Cheung C.Y., Ran A.R., Wang S., Chan V.T.T., Sham K., Hilal S., Venketasubramanian N., Cheng C.Y., Sabanayagam C., Tham Y.C. (2022). A deep learning model for detection of Alzheimer’s disease based on retinal photographs: A retrospective, multicentre case-control study. Lancet Digit. Health.

